# A Comparison of Canal-centering ability of Two Nickel-Titanium Rotary Systems with Nickel Hand Instrumentation with Stainless Steel Hand Instrumentation in 10 to 25° Curved Canals using Kuttler's Cube

**DOI:** 10.5005/jp-journals-10005-1256

**Published:** 2015-02-09

**Authors:** Anupama Swarnkar

**Affiliations:** Reader, Department of Conservative Dentistry, Mahatma Gandhi Dental College, Jaipur, Rajasthan, India

**Keywords:** Kuttler's endodontic case, Canal-centering ability, NiTi rotary instruments, Profile, Lightspeed (LSX).

## Abstract

**Aims:** The purpose of this study was to compare the canal centering abilities of rotary nickel-titanium (NiTi) files (ProFile 0.04 and 0.06 and Lightspeed LSX) and NiTi hand K-files in 10° to 25° curved canals. This was compared with the canal centering ability of stainless steel hand K-files using Kuttler's endodontic cube.

**Settings and design:** The teeth with a 10° to 25° of clinical mesiodistal curvature were used for this study. Each tooth was immersed in methylene blue dye for a period of 10 seconds, as recommended by Kuttler.

**Materials and methods:** The study sample comprised of 60 intact freshly extracted single rooted human mandibular premolars. The radiographs were traced on a tracing paper and the canal curvature was determined according to methodology introduced by Schneider.

**Statistical analysis used:** The statistical package SPSS PC + (Statistical package for social service, Version 4.01) was used for analysis.

**Results:** Overall, Lightspeed LSX instruments showed superior canal centering ability and performed better than Profile series, hand NiTi K-files and hand stainless steel K-files.

**Conclusion:** The endodontic cube can be used as an effective method for analyzing the canal-centering ability of different endodontic instruments. Both the NiTi rotary instruments showed superior canal-centering ability than NiTi and stainless steel hand K-files.

**How to cite this article:** Swarnkar A. A Comparison of Canal-centering ability of Two Nickel-Titanium Rotary Systems with Nickel Hand Instrumentation with Stainless Steel Hand Instrumentation in 10 to 25° Curved Canals using Kuttler's Cube. Int J Clin Pediatr Dent 2014;7(3):157-162.

## INTRODUCTION

Cleaning and shaping is an important phase in endo-dontic therapy. Schilder (1974) had stated that the objective of making the fnal root canal preparation confine to the general shape and direction of the original canal may be the most neglected phase of endodontic treatment, and the greatest problem lies in attempting to maintain the canal curvature in the apical region. However, ledge formation, transportation of apical foramen, and nontapered hourglass-shaped preparation are problems frequently observed after instrumentation in curved root canals.^1^ Cimis et al^2^ reported that 46% of curved canals exhibited various degrees of apical transportation following instrumentation. To overcome these inconveniences, nickel-titanium (NiTi) rotary-shaping techniques were developed more than a decade ago.^3^

Different methods have been used to evaluate the efficiency and deficiency that instruments used for root canal preparation can produce. Bramante et al in 1987^4^ addressed this issue with the introduction of a model that consisted of tooth embedded in resin, which could be sectioned horizontally into a number of slices before instrumentation and then held together by an external muffe system which was made up of plaster during instrumentation. Postinstrumentation sections could then be removed to allow image capture and compared with analyzed preinstrumentation images. But, this model had the tendency for section movement during instrumentation. Kuttler in 2001^5^ introduced a model called the endodontic cube. This allowed the observer to capture and compare the pre and postinstrumentation features of the same root canal with more precision.

The purpose of this study was to compare the canal centering abilities of rotary NiTi files (ProFile 0.04 and 0.06 and Lightspeed LSX) and NiTi hand K-files in 10° to 25° curved canals. This was compared with the canal centering ability of stainless steel hand K-files using Kuttler's endodontic cube, which was simple in use, with reduced errors, provided transverse sections and better pre and postoperative evaluation.

## MATERIALS AND METHODS

The study sample comprised of 60 intact freshly extracted single rooted human mandibular premolars. Standard access opening was prepared with a #2 round bur in a high-speed handpiece. Working length was established 1 mm short of the canal length that was determined by placing a #10 file into each canal, until it was just visible at the apical foramen. The teeth were then radiographed bucally and mesially to ensure that the canals were not obstructed and there was no second canal in the teeth. The radiographs were traced on a tracing paper, and the canal curvature was determined according to methodology introduced by Schneider.^6^ The teeth with a 10° to 25° of clinical mesiodistal curvature were used for this study. The teeth were sectioned occlusally to maintain a standard length of 12 mm for all the specimens. The apical foramen was sealed with sticky wax. Each tooth was immersed in methylene blue dye^13^ for a period of 10 seconds, as recommended by Kuttler.^5^ This will enable to clearly distinguish the external outline of the embedded tooth in each section.

**Fig. 1:**
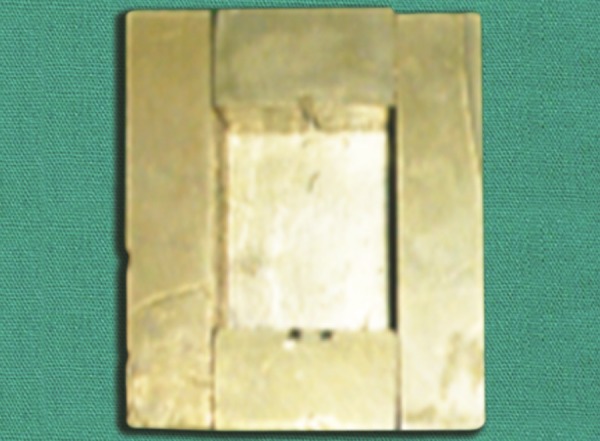
Endo Kuttler cube

**Fig. 2:**
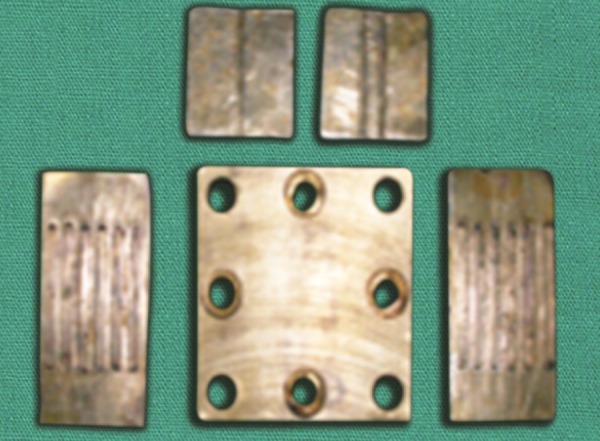
Five sections of the cube

**Fig. 3:**
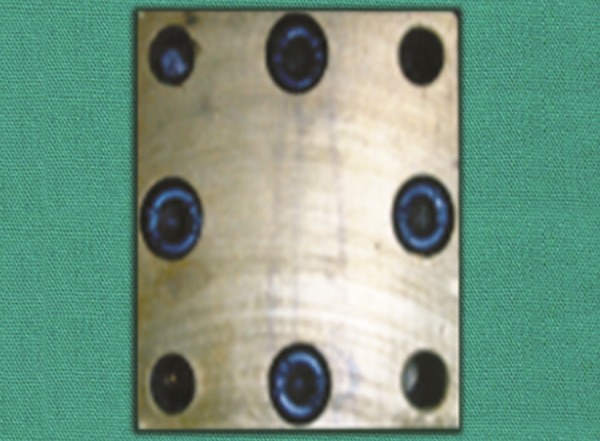
Vertical and horizontal grooves

**Fig. 4:**
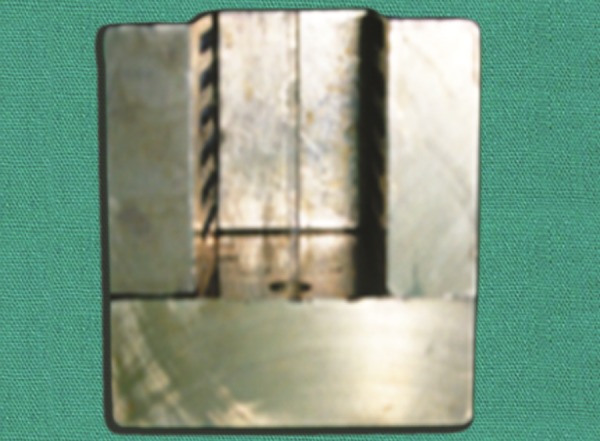
Acrylic block sectioned at two levels

**Fig. 5:**
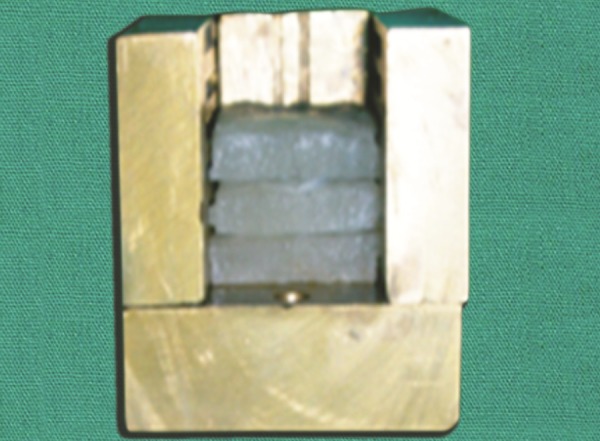
External fixation screws

**Fig. 6:**
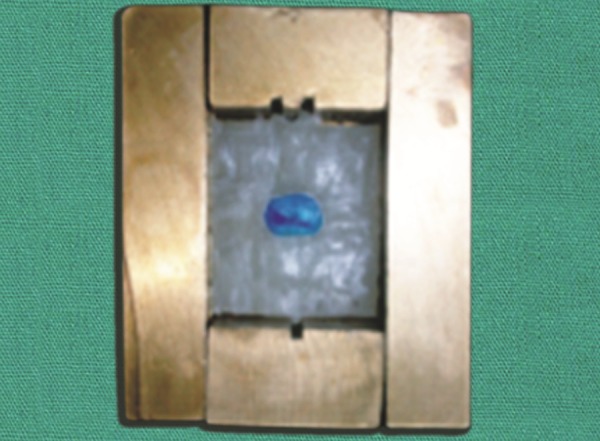
Tooth embedded in acrylic

The endodontic cube consisted of five sections that were held together by external fixation to form a roofess cube (Fig. 1). The vertical walls had horizontal grooves projecting internally by 1 mm that were machined at precise intervals of 1.5 mm (Fig. 2). They provided the internal indexing in the horizontal plane and the guides for the site at which the resin tooth model would be sectioned (Fig. 3). Two vertical sections that had longitudinal grooves to correctly orient the sections after image was captured, that increased the ease of reassembly and completed the open cube (Fig. 4). External fixation screws held the outer sections together (Fig. 5). Each tooth was embedded in acrylic resin using endodontic cube, which was placed on a laboratory vibrator (Fig. 6). After the acrylic had set, the endodontic cube was disassembled and the embedded tooth was removed from the cube. The acrylic block demonstrated equidistant horizontal grooves on opposite surfaces; whereas the remaining two opposite walls had vertical surface projection.

This created three distinct areas of analysis namely:

1. Coronal third – canal orifice
2. Middle third – 4.5 mm from canal orifice
3. Apical third – 9 mm from canal orifice

## PREINSTRUMENTATION IMAGING

Each section was placed in silicon impression material, to held each section in the same position for pre- and post-instrumentation photographs. The specimen was then photographed using camera (Nikon S3) with macro mode, which was fixed on a stand to maintain the same distance between the camera and specimens to get photos with 1:1 reproduction ratio.

Specimens were divided randomly into four experimental groups of 15 samples each.

The test groups comprised of:

- * Group I:* Specimens prepared with stainless steel K-files and Gates Glidden Drills.
- * Group II:* Specimens prepared with NiTi K-files and Gates Glidden Drills.
- * Group III:* Specimens prepared with ProFile 0.04 and 0.06 series.
- * Group IV:* Specimens prepared with Lightspeed LSX and Gates Glidden Drills.

### Group I

Group I was prepared by hand instrumentation with step back technique with anticurvature fling in a quarter-turn pull, motion, using stainless steel K-files (Mani Inc, Japan). These instruments were precurved, before their introduction in canal because of their rigidity. The canals were instrumented to working length with a size 20 file. Gates Glidden Drills (Mani Inc, Japan) sizes 2, 3 and 4 were used in coronal third of the canal, without applying apical pressure. The canals were instrumented to working length with a size 20 file. Gates Glidden Drills (Mani Inc, Japan) sizes 2, 3 and 4 were used in coronal third of the canal, without applying apical pressure. Hand instrumentation was continued to a size 30 at working length.

### Group II

Method is same as group I but specimens prepared with NiTi K-files and Gates Glidden Drills.

### Group III

Group III was prepared with ProFile 0.04 and 0.06 taper (Dentsply, Maillefer, Switzerland) following the full sequence recommended by the manufacturer.

For apical shaping, ProFile #15/0.04, #20/0.04, #20/0.06, #25/0.04, #25/0.06, and #30/0.04 were sequentially used to the working length. Profile instruments were used with a 16:1 gear reduction handpiece (X-Smart, Dentsply) at a constant speed of 150 to 300 rpm.

Canal lubrication is accomplished with RC-Prep (Premier, Philadelphia, PA, USA). Irrigation was performed with 1 ml of 2.5% NaOCl after each instrument used. A fnal fush of 5 ml of NaOCl was used after instrumentation completion.

### Group IV

Group IV was prepared with Lightspeed extra instruments (LSX). Prepare the access and fare the coronal third with Gates Glidden Drills (Mani Inc, Japan). Determine the working length and ensure canal patency with a #15 K-file. Begin with the LSX #20, continue with sequentially larger sizes until the apical part of the canal is prepared to the correct fnal apical size (FAS). This is the size that requires a firm push in the fnal apical 4 mm to advance it to working length (WL). This file is known as the master apical file (MAF) or master apical rotary (MAR). MAR was 45. With the hand-piece rotating, enter the canal and slowly advance the no. 45 LSX apically. If there is no resistance keep advancing to working length. If there is resistance (blade engages walls), pause there for a moment, and then advance to working length with a slow, continuous pushing motion. Step back from working length in 2 mm increments with sequentially larger instruments, until reaching an instrument that is 25 sizes larger than the FAS.

## IMAGE ANALYSIS

The traced outlines of the canals were superimposed and analyzed using Adobe Photoshop version 7.0. This software was used to compare the uninstrumented canal images to instrumented canal images. This software allowed measuring the distance between two points by overlying photographs of instrumented and uninstru-mented canals.

**Figs 7A to D:**
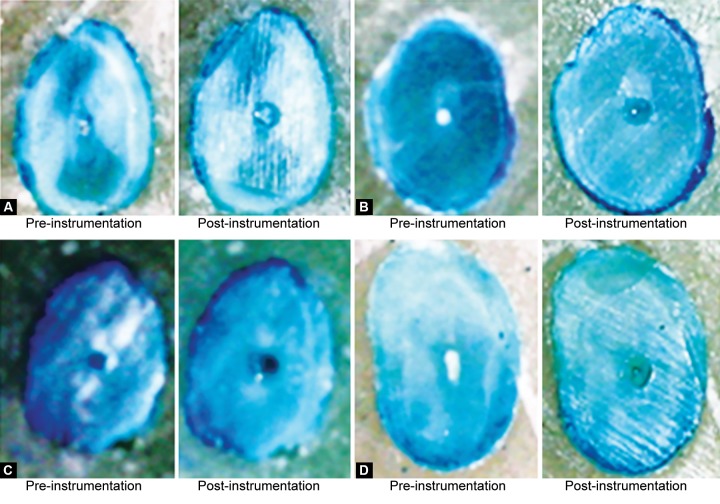
(A) Apical preparation with ss, (B) Apical preparation with Profile, (C) Apical preparation with NiTi hand files and (D) Apical preparation with Lightspeed

The distance that the canal centers moved after instrumentation was measured by overlying photographs of instrumented and uninstrumented canals in mesiodistal direction (Figs 7A to D).

The center of the canal before instrumentation was calculated by locating the center point of a rectangle outlining the greatest buccolingual and mesiodistal extent of each canal. A black dot, one pixel in size was used to denote this center point. Because circular/oval preparations were made with both sets of instruments, the center of the canal after instrumentation was determined by instrumented superimposing the best ftting circle over the preparation. In situations, where an ovoid or irregularly shaped uninstrumented canal existed, the circular instrumented canal preparation was readily distinguished from the remaining uninstrumented portion. The center point of this circle was found and marked with a white dot that was one pixel in size. The data were stored in the computer for statistical analysis.

Results obtained were tabulated and statistically analyzed. The statistical package SPSS PC+ (Statistical package for social service, version 4.01) was used for analysis. The mean values were compared by one-way ANOVA. Multiple range test by Tukey-HSD (honestly significant difference) procedures was employed to identify the significant groups. In the present study, p < 0.05 was considered as the level of significance.

## RESULTS

### Canal Center Displacement

At the coronal level, group I (0.12 ± 0.02) showed highest canal center displacement followed by group II (0.11 ± 0.02) and group III (0.10 ± 0.02 ) and IV (0.10 ± 0.01). There was no statistically significant difference between the four groups (p = 0.05<) (Table 1).

At the middle level, group I (0.14 ± 0.02) showed highest canal center displacement followed by group II (0.10 ± 0.01) and groups III (0.05 ± 0.01) and IV (0.05 ± 0.01). There was statistically significant difference between the groups (p-value of < 0.02). t-test showed that group I was statistically significant to group II, group III and group IV. There was also statistically significant difference between the groups II, III and IV. There was no statistically significant difference between the groups III and IV.

At the apical level, group I (0.16 ± 0.02) showed highest canal center displacement followed by group II (0.10 ± 0.02) and group III (0.04 ± 0.01) and group IV (0.03 ± 0.01). There was statistically significant difference between the groups (p-value of <0.02). t-test showed that group I was statistically significant to groups II, III and IV. There was also statistically significant difference between the groups II, III and IV. There was no statistically significant difference between the groups III and IV.

**Table 1:** **Table 1:** Standard deviation and range for canal center displacement of the four groups at coronal, middle and apical levels

*Instruments (groups)*		*Level*		*Scores*			
				*Mean* ± *SD*		*Range*	
1. Stainless		Coronal		0.12 ± 0.02		0.09-0.16	
steel (I)		Middle		0.14 ± 0.02		0.09-0.17	
		Apical		0.16 ± 0.02		0.13-0.20	
2. Hands		Coronal		0.11 ± 0.02		0.06-0.15	
NiTi (II)		Middle		0.10 ± 0.01		0.08-0.12	
		Apical		0.10 ± 0.02		0.07-0.14	
3. ProFile		Coronal		0.10 ± 0.02		0.06-0.17	
(III)		Middle		0.05 ± 0.01		0.03-0.07	
		Apical		0.04 ± 0.01		0.03-0.11	
4. Lightspeed		Coronal		0.10 ± 0.01		0.08-0.13	
LSX (IV)		Middle		0.05 ± 0.01		0.03-0.11	
		Apical		0.03 ± 0.01		0.02-0.06	

## DISCUSSION

It is axiomatic that ‘well-shaped canals produce well-packed canals’. Consistently producing shape is one of the strategic cornerstones in the foundation of endodontic success.

Many problems were encountered using stainless steel instruments in curved canal. Parameswaran A et al,^7^ and Coleman CT et al^8^ reported incidence of transportation, zipping and straightening of the canals using stainless steel instruments. The attention was then shifted to change of instrument design to make it more fexible and use of noncutting tips. With the introduction of more fexible stainless steel files, e.g. fexofiles, K-files and Flex-R files,^9^ the prevalence of these defects decreased, however, they were not eliminated.^10^

Walia et al^11^ introduced a new generation of instruments, wherein stainless steel was replaced by NiTi alloy. They were two to three times more elastic, and had superior resistance to fracture in clockwise torsion, when compared with similarly manufactured stainless steel files. To reduce the operator's fatigue and save time, many rotary NiTi systems were developed.

At the coronal level, there was no significant difference among all the four groups for canal center movement. At the middle level, there was significant difference between both hand stainless steel and hand NiTi K-file groups and ProFile and Lightspeed LSX rotary systems. There was significant difference between hand stainless steel and hand NiTi K-file groups, but no significant difference between ProFile and Lightspeed LSX systems.

Coleman^12^ compared the stainless steel hand K-files with that of NiTi hand files using Bramante methodology. He reported a mean canal center movement of 0.24 ± 0.05 mm for stainless steel hand K-files and 0.13 ± 0.07 for NiTi hand files at the apical sections toward mesial side. At the middle section, it was 0.18 ± 0.10 mm and 0.12 ± 0.05 for stainless steel hand K-files and NiTi hand files respectively. Our study has also observed similar values for stainless steel hand K-files and NiTi hand K-files.

At the coronal level, all the four groups showed results that were not statistically significantly different. This showed that in straighter portion of the canal, both hand stainless steel and hand and rotary NiTi systems could perform equally well. Studies^9,13^ have reported similar results comparing different stainless steel and NiTi systems.

In the middle level, all the four groups showed results that were statistically significant. Hand stainless steel K-files transported the canal. There was statistically significant difference between hand stainless steel K-files and hand NiTi K-files that is according to the studies.^13,14^ ProFiles and Lightspeed instruments performed signif-cantly better than the hand stainless steel and hand NiTi K-files. There was no statistically significant difference between ProFiles and Lightspeed instruments.^15^ The superior performance of ProFiles and Lightspeed instruments may be related to several factors: the instrument design, NiTi alloy or the Reaming technique.^14,15^

In the apical level, the hand stainless steel K-files caused more transportation than both the rotary NiTi systems. This result is also expected because of the rigidity of stainless steel. Hand NiTi K-files maintained canal center better than hand stainless steel K-files and it was statistically significant. Both ProFiles and Lightspeed instruments maintained canal curvature well and there was no statistically significant difference between the two.^15^ Hand NiTi K-files showed significant canal center movement in comparison to ProFiles and Lightspeed instruments. The increased fexibility of the NiTi files and the safety tips with rounded transition angel was discussed as the main factor for the superior shaping ability of ProFiles and Lightspeed instruments.

In our study, the noncutting tip, their cross-sectional design, along with their fexibility could be the reasons for the hand and rotary NiTi system to remain more centered than the stainless steel file. But, certain deviations from canal anatomy have been reported with the use of hand stainless steel instruments. ProFiles and Lightspeed instruments and hand NiTi K-files performed good in comparison with hand stainless steel K-files.^16-18^ In our study, Lightspeed instruments are good in maintaining canal curvature because of its spade design in comparison to the ‘U’ design of ProFile, that makes the instrument more fexible, eliminates futes that are flled with debris and reduces blades cutting surfaces, thereby enhancing the cutting efficiency.

## CONCLUSION

The endodontic cube can be used as an effective method for analyzing the canal centering ability of different endo-dontic instruments. Both the NiTi rotary instruments (ProFile series and Lightspeed LSX) showed superior canal centering ability than NiTi and stainless steel hand K-files. Overall, Lightspeed LSX instruments showed superior canal centering ability and performed better than ProFile series, hand NiTi K-files and hand stainless steel K-files. However, results obtained by Lightspeed LSX and ProFile instrumentation did not show a very significant variation.

All the test specimens irrespective of the instrumentation technique employed could not demonstrate perfect canal centering ability.
